# Choosing between Enoxaparin and Fondaparinux for the management of patients with acute coronary syndrome: A systematic review and meta-analysis

**DOI:** 10.1186/s12872-017-0552-z

**Published:** 2017-05-08

**Authors:** Pravesh Kumar Bundhun, Musaben Shaik, Jun Yuan

**Affiliations:** 1 Institute of Cardiovascular Diseases, the First Affiliated Hospital of Guangxi Medical University, Nanning, Guangxi 530027 People’s Republic of China; 2 Department of Paediatrics, Pragati Children’s Hospital, Ch Pet, Andhra Pradesh India; 3 Department of Cardiology, The People’s Hospital of Guangxi Zhuang Autonomous Region, Nanning, Guangxi 530021 China

**Keywords:** Enoxaparin, Fondaparinux, Acute coronary syndrome, Major bleeding, Minor bleeding, Heparin

## Abstract

**Background:**

Enoxaparin and Fondaparinux are potential anticoagulants which are used peri-operatively in the management of patients with Acute Coronary Syndrome (ACS). We aimed to compare the adverse clinical outcomes which are associated with the use of these anticoagulants in patients who were treated for ACS.

**Methods:**

Online databases (PubMed/Medline, EMBASE, Cochrane library) were searched for studies which compared differences in clinical outcomes observed with the use of enoxaparin and fondaparinux in patients who were treated peri-operatively for ACS. Statistical analysis was carried out by Revman 5.3 software with odds ratio (OR) and 95% confidence intervals (CI) as the analytical parameters.

**Results:**

Seven studies with a total number of 9618 patients (mainly composed of non-ST elevated myocardial infarction/NSTEMI) were included. This analysis showed mortality to be similarly observed between enoxaparin and fondaparinux with OR: 1.05, 95% CI: 0.67–1.63; *P* = 0.84. Myocardial infarction (MI) and stroke were also not significantly different throughout different follow up periods. However, minor, major and total bleeding were significantly lower with fondaparinux (OR: 0.40, 95% CI: 0.27–0.58; *P* = 0.00001), (OR: 0.46, 95% CI: 0.32–0.66; *P* = 0.0001) and (OR: 0.47, 95% CI: 0.37–0.60; *P* = 0.00001) respectively during the 10-day follow up period. Even during a follow up period of 30 days or a midterm follow up, major and minor bleeding still significantly favored fondaparinux in comparison to enoxaparin.

**Conclusion:**

In patients who were treated for ACS, fondaparinux might be a better choice when compared to enoxaparin in terms of short to midterm bleeding events. This result was mainly applicable to patients with NSTEMI. However, due to a limited number of patients analyzed, further larger randomized trials should be able to confirm this hypothesis.

## Background

Enoxaparin and Fondaparinux are potential anticoagulants which are used peri-operatively in the management of patients with Acute Coronary Syndrome (ACS) [1]. The Fifth Organization to Assess Strategies in Ischemic Syndromes (OASIS 5) trial showed fondaparinux to reduce the rate of major bleeding and net clinical benefit including death, Myocardial Infarction (MI), stroke and major bleeding in comparison to enoxaparin [2]. However, results from the French Registry of ST segment elevation and non-ST segment elevation MI (FAST-MI) 2010 showed a similar rate of bleeding and mortality between fondaparinux and enoxaparin [3]. Even though, FAST-MI cannot be compared to the OASIS 5 trial which consisted of a very large number of patients, we would still like to confirm the results through a meta-analysis by comparing the adverse clinical outcomes which were observed with enoxaparin and fondaparinux in patients who were treated for ACS.

## Methods

### Data sources and search strategy

Online databases (PubMed/Medline, EMBASE, Cochrane library) were searched for studies (English publications) which compared differences in clinical outcomes observed with the use of enoxaparin and fondaparinux in patients who were treated peri-operatively for ACS by using the searched terms ‘enoxaparin and fondaparinux and acute coronary syndrome’. Later in this search process, the terms ‘percutaneous coronary intervention, heparin’ and the abbreviations ‘ACS and PCI’ were also used. Reference lists of suitable articles were also reviewed for relevant publications.

### Inclusion criteria

Studies were included if they satisfied certain major criteria which were:

- They were randomized controlled trials or observational studies comparing enoxaparin with fondaparinux in patients who were treated for ACS or in patients who were undergoing percutaneous coronary intervention (PCI).
- They reported adverse outcomes (cardiovascular and bleeding outcomes) as their clinical endpoints.
- They involved relevant data which could be used in this current analysis.

### Exclusion criteria

Studies were excluded if they did not satisfy certain major criteria which were:

- They were systematic reviews, meta analyses, case studies or letter to editors.
- They did not include patients with ACS.
- They did not report the previously mentioned clinical outcomes.
- They were duplicates of the same study or they were associated with the same trial or cohort.

### Types of participants

This analysis mainly included patients with non-ST segment elevated myocardial infarction (NSTEMI), patients with unstable angina (UA) and a small percentage of patients with ST segment elevated myocardial infarction (STEMI).

### Definitions, outcomes and follow ups

Endpoints which were assessed included:

- Mortality;
- MI;
- Stroke;
- Minor bleeding: any type of minor bleeding;
- Major bleeding: any type of major bleeding;
- All bleeding (major and minor bleeding combined) including thrombolysis in myocardial infarction (TIMI) defined major and minor bleeding [4].

The follow up periods were classified as:

- Less than 10 days (including in-hospital follow up period);
- 30 days follow up period;
- Mid-term follow up (6 months to 1 year) period.

These outcomes and follow up periods have been summarized in Table 1.

**Table 1:** Reported Outcomes and follow up periods

Studies	Reported outcomes	Follow up period	Types of participants
FAST MI [3]	Death, MI, stroke, TIMI major and minor bleeding	In-hospital	NSTEMI
OASIS 5 [2]	Death, MI, stroke, TIMI major bleeding, total bleeding, major bleeding	9 days, 30 days, 6 months	NSTEMI, UA
Schiele 2010 [14]	Death, bleeding	30 days	STEMI, NSTEMI, UA
Shah 2014 [15]	Death, bleeding	9 days, 30 days	UA, NSTEMI
Zhao 2015 [12]	Death, MI, major bleeding, minor bleeding, stroke	7 days, 30 days, 6 months	NSTEMI
Zhao 2016 [16]	Death, MI, stroke, major bleeding, all bleeding	30 days, 1 year	STEMI, NSTEMI, UA
Soeiro 2016 [10]	Death, MI, major bleeding, stroke	In-hospital	NSTEMI

*Abbreviations*: *MI* myocardial infarction, *TIMI* thrombolysis in myocardial infarction, *STEMI* ST elevated myocardial infarction, *NSTEMI* non-ST elevated myocardial infarction, *UA* unstable angina

### Data extraction and review

Information and data including the name of authors, year of article publication, period of patients’ enrollment, number of patients collected from each group (enoxaparin and fondaparinux), type of study (RCT or observational study), baseline characteristics of the patients, the outcomes reported, the follow up periods, the medications used by the patients, and the number of events reported with enoxaparin and fondaparinux respectively, were independently collected/extracted by two authors (PKB and MS). Disagreements were resolved by discussion with the third author (JY). Since this is a meta-analysis, the PRISMA guideline [5] was followed and the bias risk among the trials was assessed with reference to the features which have been stated in the Cochrane Collaboration [6]. All the trials were rated as having a ‘low to moderate’ risk of bias.

### Statistical analysis

The statistical analysis was carried out by Revman 5.3 software with odds ratio (OR) and 95% confidence intervals (CI) as the analytical parameters. Two simple statistical methods were used to assess heterogeneity [7] across the studies namely the Q statistic test and the I^2^ test.

A *P* value of less or equal to 0.05 was considered as statistically significant whereas a *P* value greater than 0.05 was insignificant in this analysis.

On the other hand, a fixed effects model or a random effects model was used during the analysis depending on the value of I^2^. A fixed effects model was recommended if an I^2^ value <50% was obtained, whereas if the I^2^ value was >50%, a random effects model was recommended.

In addition, this I^2^ value was also used to predict heterogeneity. The lower the I^2^ value, the lesser would be the heterogeneity whereas heterogeneity would increase with an increasing I^2^ value.

Sensitivity analyses were also carried out by excluding each study one by one and a new analysis was carried out each time.

Publication bias was visually observed by analyzing the funnel plots which were generated through the RevMan software. Since this analysis did not involve a large volume of studies (only 7 studies were available), asymmetry of the funnel plots was sufficient to represent publication bias. Normally, if the total number of studies which were included was more than 10, other methods would have been more appropriate.

Ethical approval was not required for this type of study.

## Results

### Search results

Seven hundred and eighty-seven (787) articles were obtained using the above mentioned searched terms from electronic databases. After a careful check of the abstracts and titles by the same two authors (PKB and MS), 764 articles were eliminated (not related to this current idea). Twenty-three (23) full-text articles were assessed for eligibility. Among these 23 articles, further studies were eliminated because they were:

- Systematic reviews, case studies or letter to editors (2);
- Articles with data which were irrelevant for this current meta-analysis (1);
- Articles which did not report adverse clinical outcomes as their endpoints (4);
- Duplicates or studies which were associated with the same trials or cohorts (9).

**Fig. 1:**
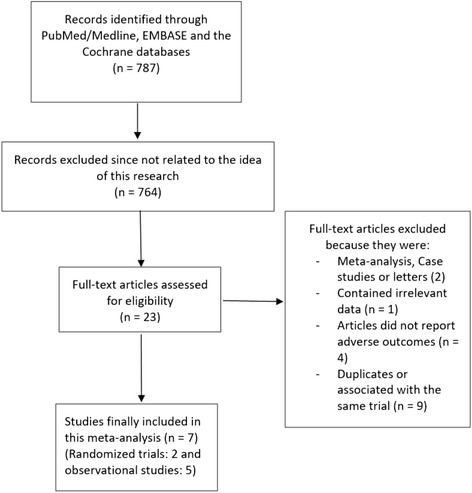
Flow diagram representing the study selection

Finally, 7 studies were selected and included in this analysis (Fig. 1).

### General features of the studies which were included

Five (5) observational studies and 2 randomized trials were included with a total number of 9618 patients (6587 patients were treated with enoxaparin and 3031 patients were treated with fondaparinux). Regions of patients’ enrollment included France, China, Brazil, and Canada with an enrollment period ranging between years 2003 to 2015. The general features of the studies which were included have been listed in Tables 2 and 3.

**Table 2:** General features of the studies which were included

Studies	Type of study	Region	Patients’ enrollment year	Total no of patients in Enoxaparin group (n)	Total no of patients in Fondaparinux group (n)
FAST MI	Observational	France	2010	1027	240
OASIS 5	RCT	Canada	2003–2005	1420	1414
Schiele2010	Observational	France	2006–2007	1418	301
Shah2014	RCT	-	2010	90	90
Zhao2015	Observational	China	2011–2012	232	229
Zhao2016	Observational	China	2010–2012	453	422
Soeiro2016	Observational	Brazil	2010–2015	1947	335
Total no of patients (n)				6587	3031

*Abbreviations*: *RCT* randomized controlled trials

**Table 3:** Procedures and duration of therapy

Studies	Procedures	PCI (%)	Type of CAD	Duration of therapy
	Eno/Fond	Eno/Fond	Eno/Fond	Eno/Fond
FAST MI	PCI	69.0/69.0	NSTEMI	2 days
OASIS 5	PCI	100/100	NSTEMI, UA	2–8 days
Schiele2010	PCI	84.0/72.0	STEMI, NSTEMI, UA	In hospital period
Shah2014	PCI	100/100	UA, NSTEMI	In hospital period
Zhao2015	PCI	100/100	NSTEMI	2–8 days
Zhao2016	PCI	100/100	STEMI, NSTEMI, UA	3–7 days
Soeiro2016	PCI	100/100	NSTEMI	In hospital period

*Abbreviations*: *PCI* percutaneous coronary intervention, *Eno* enoxaparin, *Fond* fondaparinux, *CAD* coronary artery disease, *STEMI* ST segment elevated myocardial infarction, *NSTEMI* non-ST segment elevated myocardial infarction, *UA* unstable angina

### Baseline features of the studies which were included

The baseline features (Table 4) were as follow: mean age 57.3–67.0 years, majority of the patients were male patients, other co-morbidities and smoking history were also reported in both groups. Overall, there were no significant differences in the baseline features between patients who were treated with enoxaparin and fondaparinux.

**Table 4:** Baseline features of the studies which were included

Studies	Mean age	Males (%)	Ht (%)	Ds (%)	Cs (%)	DM (%)
	E/F	E/F	E/F	E/F	E/F	E/F
FAST MI	67.0/66.5	71.0/72.0	58.0/66.0	45.5/52.5	27.0/32.0	23.0/25.0
OASIS 5	64.5/64.6	69.1/71.7	-	-	-	23.1/23.5
Schiele2010	-	71.0/66.0	52.0/57.0	49.0/55.0	32.0/31.0	21.0/20.0
Shah2014	**-**	77.8/74.4	55.6/55.6	-	58.9/55.6	41.1/38.9
Zhao2015	59.8/60.1	73.3/76.9	65.5/66.8	-	68.5/68.1	39.6/37.1
Zhao2016	58.2/57.3	78.2/76.3	59.5/57.2	-	53.0/52.1	20.3/22.8
Soeiro2016	61.8/61.0	62.6/65.7	73.6/67.8	51.2/48.9	30.5/24.2	46.9/55.8

*Abbreviations*: *E* enoxaparin, *F* fondaparinux, *Ht* hypertension, *Ds* dyslipidemia, *Cs* current smoker, *DM* diabetes mellitus

The OASIS 5 trial had a large number of patients when compared to the other studies. However, in order for the current results not to be influenced by the results obtained in the OASIS 5 trial, the proportion of patients obtained from the OASIS 5 trial was reduced in order to adjust to this current analysis. To be more clear, only the percentage of female patients (20–30%) from the trial were included when analyzing the 30-day outcomes and only 11 to 12% of patients were included when assessing bleeding events. It should be noted that the total number of patients which were extracted was not reduced. For example, ‘10 events of death out of 100 patients’ was represented as ‘1 event of death out of 10 patients’. Therefore, it is basically the same thing.

Table 5 summarized other anti-platelet and anti-coagulant medications which were used by the patients. It can clearly be seen that almost all the patients were also being treated by aspirin and clopidogrel. Glycoprotein IIb/IIIa, prasugrel and unfractionated heparin were also being used by certain patients.

**Table 5:** Other anti-platelet and anticoagulant medications which were used by the participants

Studies	Aspirin	Clopidogrel	Glycoprotein IIb/IIIa	Prasugrel	U.heparin
	E/F	E/F	E/F	E/F	E/F
FAST MI	98.0/98.0	90.5/92.0	26.5/34.0	15.0/15.0	36.0/60.0
OASIS 5	98.9/98.6	92.3/91.1	38.8/40.4	-	-
Schiele2010	99.0/99.0	98.0/99.0	58.0/68.0	-	-
Zhao2015	96.5/96.1	100/100	-	-	100/100
Zhao2016	99.8/99.3	75.3/80.1	-	-	-
Soeiro2016	97.8/98.5	67.9/65.4	16.1/5.80	-	-

*Abbreviations*: *E* enoxaparin, *F* fondaparinux, data were represented in terms of %

### Less than 10-days follow up period (enoxaparin versus fondaparinux)

**Fig. 2:**
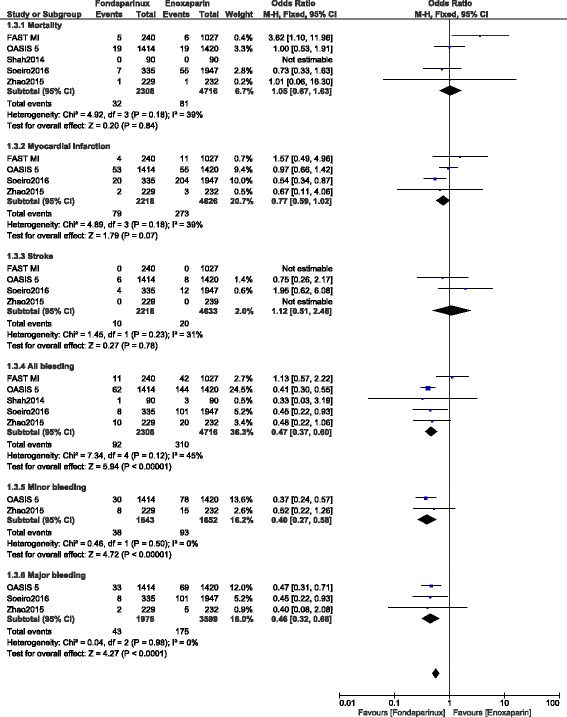
Enoxaparin versus fondaparinux (10-day follow up)

Results of this analysis showed that mortality was similarly observed between enoxaparin and fondaparinux with OR: 1.05, 95% CI: 0.67–1.63; *P* = 0.84. MI and stroke were also not significantly different with OR: 0.77, 95% CI: 0.59–1.02; *P* = 0.07 and OR: 1.12, 95% CI: 0.51–2.46; *P* = 0.78 respectively during this 10-day period. However, minor, major and total bleeding were significantly lower with fondaparinux (OR: 0.40, 95% CI: 0.27–0.58; *P* = 0.00001), (OR: 0.46, 95% CI: 0.32–0.66; *P* = 0.0001) and (OR: 0.47, 95% CI: 0.37–0.60; *P* = 0.00001) respectively. Results for this 10-day follow up period have been represented in Fig. 2.

**Fig. 3:**
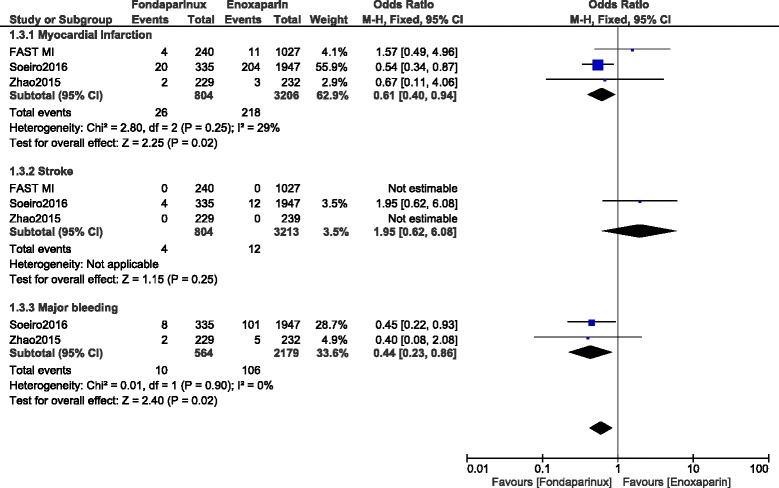
Enoxaparin versus fondaparinux using data obtained only from observational studies (10-day follow up)

When observational data were separately analyzed, MI and major bleeding significantly favored fondaparinux with OR: 0.61, 95% CI: 0.40–0.94; *P* = 0.02 and OR: 0.44, 95% CI: 0.23–0.86; *P* = 0.02 respectively (Fig. 3).

**Fig. 4:**
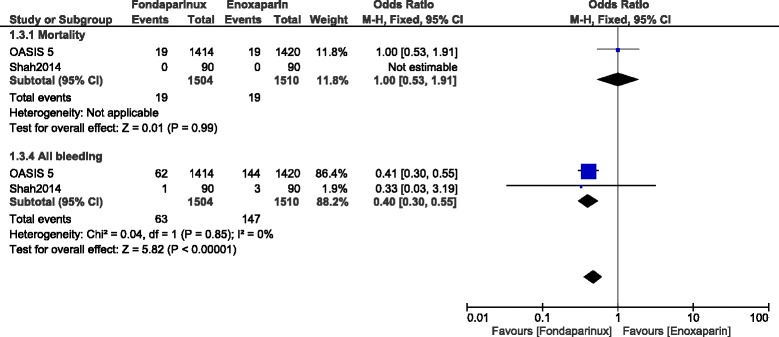
Enoxaparin versus fondaparinux using only randomized patients (10-day follow up)

However, when randomized data were separately analyzed, mortality was not significantly different between these two drugs with OR: 1.00, 95% CI: 0.53–1.91; *P* = 0.99 whereas total bleeding still significantly favored fondaparinux with OR: 0.40, 95% CI: 0.30–0.55; *P* = 0.00001 (Fig. 4).

### 30-days follow up period (enoxaparin versus fondaparinux)

**Fig. 5:**
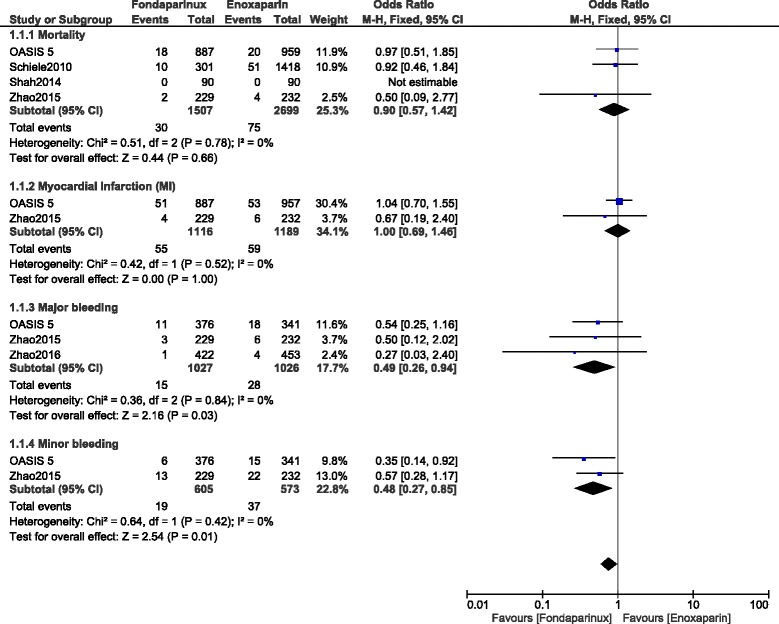
Enoxaparin versus fondaparinux (30-day follow up)

During this 30-days follow up period, mortality and MI were not significantly different with OR: 0.90, 95% CI: 0.57–1.42; *P* = 0.66 and OR: 1.00, 95% CI: 0.69–1.46; *P* = 1.00 respectively. However, major and minor bleeding significantly favored fondaparinux with OR: 0.49, 95% CI: 0.26–0.94; *P* = 0.03 and OR: 0.48, 95% CI: 0.27–0.85; *P* = 0.01 respectively. Results for the 30-days follow up period have been represented in Fig. 5.

**Fig. 6:**
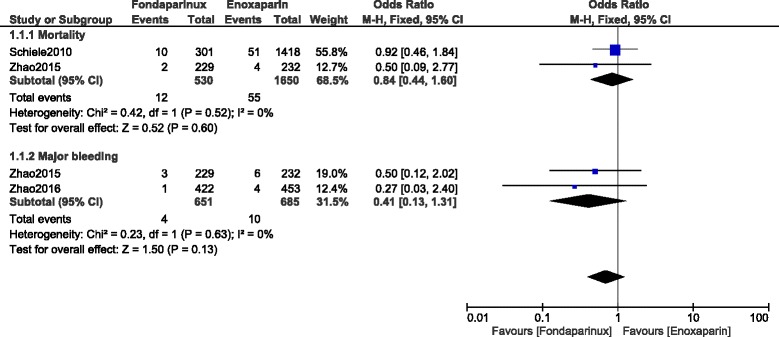
Enoxaparin versus fondaparinux using data obtained only from observational studies (30-day follow up)

When observational data were separately analyzed, mortality was not significantly different with OR: 0.84, 95% CI: 0.44–1.60; *P* = 0.60. However, even if major bleeding favored fondaparinux with OR: 0.41, 95% CI: 0.13–1.31; *P* = 0.13, the result was not statistically significant (Fig. 6).

### Midterm follow up period (enoxaparin versus fondaparinux)

**Fig. 7:**
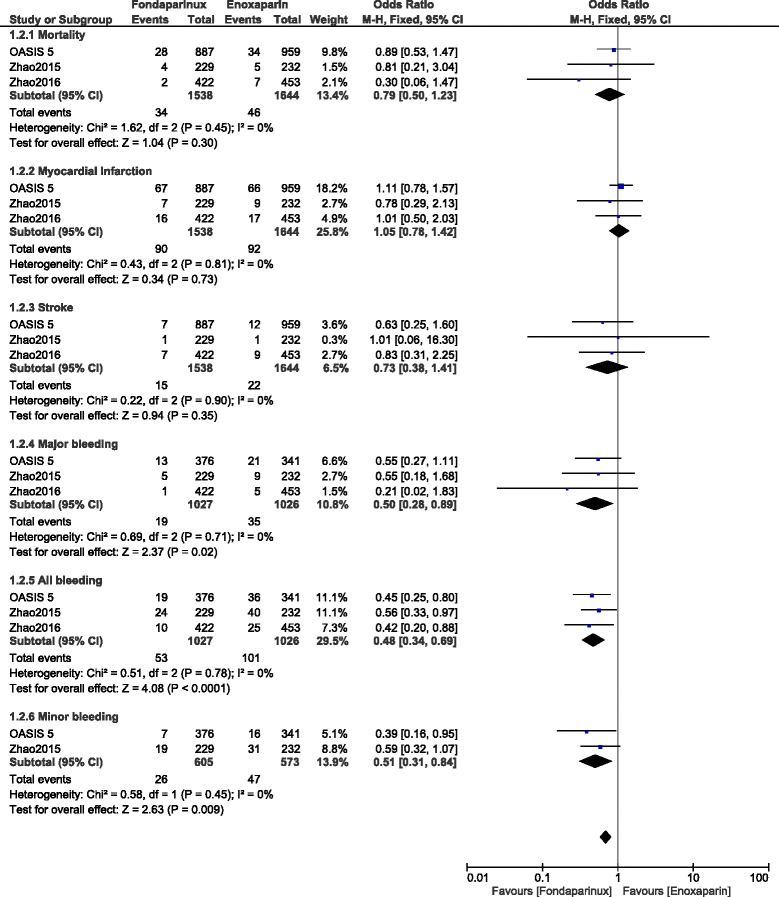
Enoxaparin versus fondaparinux (mid-term follow up)

During this midterm follow up period, mortality, MI, and stroke were still not significantly different with OR: 0.79, 95% CI: 0.50–1.23; *P* = 0.30, OR: 1.05, 95% CI: 0.78–1.42; *P* = 0.73 and OR: 0.73, 95% CI: 0.38–1.41; *P* = 0.35 respectively. However, major, minor and total bleeding were significantly lower with fondaparinux with OR: 0.50, 95% CI: 0.28–0.89; *P* = 0.02, OR: 0.51, 95% CI: 0.31–0.84; *P* = 0.009 and OR: 0.48, 95% CI: 0.34–0.69; *P* = 0.0001 respectively (Fig. 7).

**Fig. 8:**
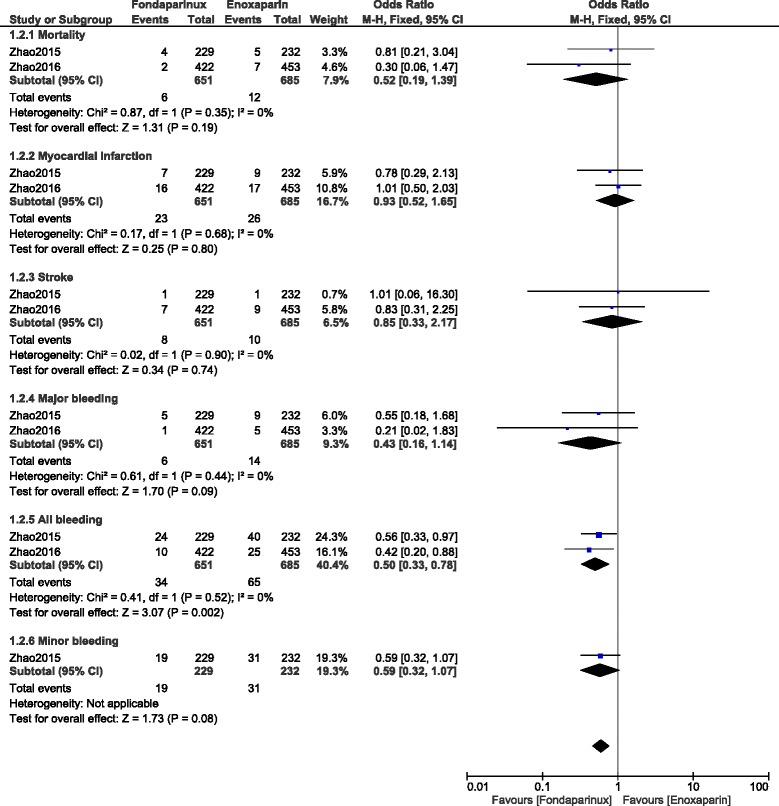
Enoxaparin versus fondaparinux using data obtained only from observational studies (mid-term follow up)

When observation studies were separately analyzed, mortality, MI and stroke were not significantly different with OR: 0.52, 95% CI: 0.19–1.39; *P* = 0.19, OR: 0.93, 95% CI: 0.52–1.65; *P* = 0.80 and OR: 0.85, 95% CI: 0.33–2.17; *P* = 0.74 respectively. However, even if major bleeding favored fondaparinux with OR: 0.43, 95% CI: 0.16–1.14; *P* = 0.09, the result was not statistically significant but total bleeding was significantly lower with fondaparinux with OR: 0.50, 95% CI: 0.33–0.78; *P* = 0.002 (Fig. 8).

### Sensitivity analysis

Sensitivity analyses were carried out. During the 30-days follow up period, excluding study Zhang2016 resulted in a non-significant result associated with major bleeding with OR: 0.53, 95% CI: 0.27–1.04; *P* = 0.06. During the midterm follow up period, excluding trial OASIS 5 resulted in a non-significant major bleeding with OR: 0.43, 95% CI: 0.16–1.14; *P* = 0.09. For the other results, no significant change was observed.

In addition, when patients with STEMI were excluded, and an analysis was conducted based on patients with NSTEMI, major and minor bleeding were still significantly higher with enoxaparin, with OR: 0.46, 95% CI: 0.32–0.66; *P* = 0.0001 and OR: 0.40, 95% CI: 0.27–0.58; *P* = 0.00001 respectively during a 10-day follow up period. During a 30-day follow up period, only minor bleeding was significantly higher with enoxaparin, with OR: 0.48, 95% CI: 0.27–0.85; *P* = 0.01. In addition, during the mid-term follow up, major and minor bleeding were both significantly higher with enoxaparin, with OR: 0.55, 95% CI: 0.30–0.99; *P* = 0.05 and OR: 0.51, 95% CI: 0.31–0.84; *P* = 0.009 respectively in these patients with NSTEMI.

### Publication bias

**Fig. 9:**
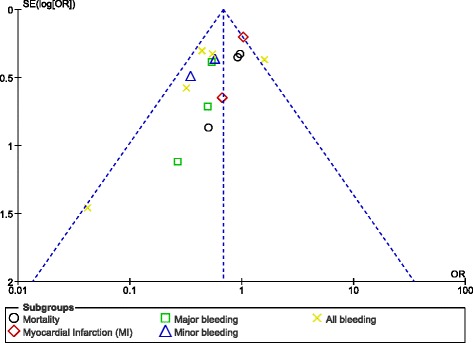
Funnel plot representing publication bias

**Fig. 10:**
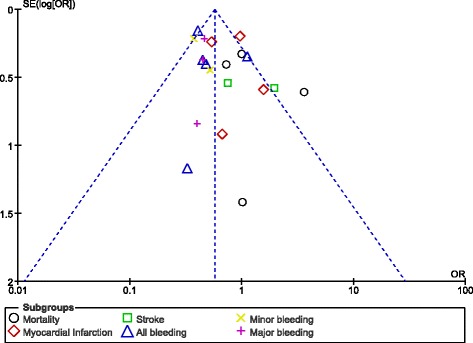
Funnel plot representing publication bias

**Fig. 11:**
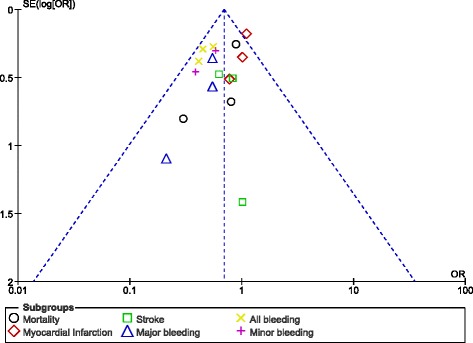
Funnel plot representing publication bias

This current analysis included of a total number of 7 studies (2 randomized and 5 observational studies) that assessed all the clinical endpoints with minimal evidence of publication bias which was visually observed through the funnel plots obtained (Figs. 9, 10, 11).

These funnel plots represented minimal publication bias. Possible reasons might be the presence of observational studies, and the selection of only English publications for this analysis.

## Discussion

This analysis aimed to compare enoxaparin with fondaparinux. Current results showed both anticoagulants to have similar mortality and stroke rates. However, fondaparinux was associated with a significantly lower major and minor bleeding when compared to enoxaparin in majority of the bleeding subgroups which were analyzed.

Surprisingly, even if the use of glycoprotein IIb/IIIa was more in patients who used fondaparinux, a low bleeding was observed with this anticoagulant when compared to enoxaparin.

At this stage, it would be interesting to know the mechanisms of action of fondaparinux and enoxaparin. Fondaparinux is a factor Xa inhibitor and does not inhibit thrombin (IIa) [8]. Enoxaparin on the other hand, binds to antithrombin to form a complex molecule that can irreversibly inactivate clotting factor Xa and it has less activity against thrombin [9]. This is how these two anticoagulants work.

The Brazilian Registry Data which was a multicentered retrospective observational study including 2282 patients also supported the result of this current analysis which is similar to recently published data in international literature showing the superiority of fondaparinux to enoxaparin in the Brazilian population in terms of bleeding outcomes following PCI (40.1% of the patients in the fondaparinux group and 35.1% of patients in the enoxaparin group underwent PCI) [10].

Similarly, the OASIS 5 trial which was a double blinded randomized trial comparing fondaparinux with enoxaparin in 6238 patients who underwent PCI showed reduced bleeding events to be associated with fondaparinux without any increase in mortality [2].

On the other hand, the OASIS 6 trial (12,092 patients obtained from 447 hospitals in 41 countries around the globe) which compared fondaparinux with placebo or unfractionated heparin in patients with STEMI showed that in patients who were not undergoing PCI, the former was associated with a lower mortality and re-infarction without increasing stroke or bleeding events [11]. However, our analysis showed no significant difference in mortality or stroke, but with significantly lower bleeding events following invasive procedures when fondaparinux was compared to enoxaparin. Could it be the invasive procedure which contributed to these different results obtained from OASIS 5 and 6?

To provide an answer to this question, a small Chinese study with NSTEMI patients who underwent PCI, showed no statistically significant difference in bleeding outcomes between fondaparinux and enoxaparin. However, it should not be ignored that both groups were also additionally treated with tirofiban [12] and the total number of patients enrolled was less when compared to the OASIS 5 and 6.

Moreover, the French cohort of NSTEMI patients who were predominantly managed invasively did not show fondaparinux to be superior to enoxaparin in terms of bleeding outcomes [3]. However, similar to the results of this analysis, mortality was not significantly different between these two anticoagulants. Nevertheless, it was shown that during the propensity score matched cohorts, a lower number of patients used aspirin or clopidogrel before admission.

Finally, the fact that several factors such as co-morbidities, age, use of anti-platelets and anticoagulants, as well as the dosage and whether these blood thinning agents were used before and after admission and the effect of plaque should all be taken into consideration when assessing bleeding risk and other adverse clinical outcomes in such patients. For example, a recent meta-analysis showed thin-cap fibro-atheroma to be highly associated with culprit plaque rupture [13]. This prevalence was higher in patients with STEMI compared to patients with NSTEMI or unstable angina. Therefore, clinical outcomes might vary from study to study due to the influence of such factors.

### Novelty

This analysis is new in several ways:

- It is among the first meta-analyses comparing enoxaparin with fondaparinux in patients who were being treated for ACS.
- This interesting idea is very important clinically.
- A low to moderate level of heterogeneity was observed in several of the subgroups analyzing the outcomes, which might be a positive aspect of this analysis.
- Randomized patients and patients obtained from observational studies were combined as well as separately analyzed.

### Limitations

Limitations were as follow:

- Due to the limited number of patients analyzed, the results might not be very accurate.
- This analysis concerned mainly patients suffering from NSTEMI.
- Classification of bleeding events was vast. Bleeding should be classified as TIMI defined bleeding, BARC defined bleeding and ACUITY defined bleeding and then analyzed.
- Use of other anti-platelets and anticoagulants and other factors might have influenced the results which were obtained.

## Conclusion

In patients who were treated for ACS, fondaparinux might be a better choice when compared to enoxaparin in terms of short to midterm bleeding events. This result was mainly applicable to patients with NSTEMI. However, due to a limited number of patients analyzed, further larger randomized trials should be able to confirm this hypothesis.

## Abbreviations

ACS: acute coronary syndrome
RCTs: randomized controlled trials
PCI: percutaneous coronary intervention
